# Supplementary material for: Neurologic Music Therapy in Geriatric Rehabilitation: A Systematic Review
Source: Healthcare (Basel). 2022 Oct 31;10(11):2187. doi: 10.3390/healthcare10112187 (PMC9690210; doi:10.3390/healthcare10112187)
Supplement: Supplementary file 1 [file healthcare-10-02187-s001.zip › File S3_Characteristics of studies qualifying for review.pdf]

Characteristics of studies qualifying for review (N = 8).

No.	Author (year)	Participants	Number of participants	NMT technique	Duration of intervention	Assessment	Main results	Study design
1.	Capato et al. (2020)	Parkinson's disease	35	Multimodal balance training supported by RAS	5 weeks	H&Y; MMSE; MBEST; BBS; TUG; TUG-dual task condition; Rapid Turns Test; N-FOGQ; MDS-UPDRS part 2 and part 3; FES-I	Physiotherapy with a specific protocol of exercises with RAS can improve balance performance in PD patients in advanced disease stages and mild cognitive decline.	RCT
2.	Calabrò et al. (2019)	idiopathic Parkinson's disease	50	RAS	8 weeks	Functional Gait Assessment; Tinetti Falls Efficacy Scale; UPDRS; EEG	RAS may be a useful add-on gait rehabilitation strategy in PD, as auditory cueing can specifically target motor cortical beta frequency range synchrony during steady-state treadmill walking in patients with PD.	RCT<br><br>registration trial: NCT03434496
3.	Braun Janzen et al. (2019)	idiopathic Parkinson's disease	37	RAS	1 hour	H&Y; MDS-UPDRS; Likert Scale; BBS; 9-HPT	This research study supports the hypothesis that rhythmic priming is possible across effector systems by demonstrating that RAS training of finger movements had immediate effects on the gait velocity and cadence of patients with PD.	RCT<br><br>Reference number: 10-1752H

4.	Lee et al. (2018)	Stroke patients	44	RAS	6 weeks	Gait analysis system OptoGait; LogitechWebcam Pro 9000; TUG; BBS; FMA	Compared to standard gait training, a 6-week programme including gait training with bilateral RAS proved to be more effective with regard to the symmetry of gait, gait ability, balance and function of lower limbs in stroke patients.	RCT<br><br>registration trial: SYUIRB2012-059
5.	Thaut et al. (2019)	Parkinson's disease	60	RAS	24 weeks	H&Y; MMSE; BBS; TUG; FES; Fall Index; a computerized stride analyser system (B&L Engineering) with a 4-camera (3D) VICON-MOTUS video motion analysis system	RAS training significantly reduced the number of falls in Parkinson's disease patients and modified key gait parameters, such as velocity and stride length.	RCT<br><br>registration trial: NCT03316365
6.	Murgia et al. (2018)	Parkinson's disease	32	RAS	5 weeks	MMSE;<br>FAB; a motion capture Smart-D system; Smart Analyzer; Modified H&Y; UPDRS; FIM; Tinetti test; SPPB; GDS; PDQ-8; FES; ABC; FOGQ	The results suggest that ecological RAS is equally effective compared to artificial RAS. Future studies should further investigate the role of ecological RAS, on the basis of information revealed by our exploratory	RCT<br><br>registration trial: NCT03228888

							analyses.	
7.	Mainka et al. (2018)	Stroke patients	35	RAS	4 weeks	FGS;<br>LOC;<br>3MWT;<br>IEB	The study provides the first evidence for a higher efficacy of RAS-treadmill training in comparison to the standard approaches (i.e., treadmill training and neurodevelopmental treatment) in restoring functional gait in SPs. The results support the implementation of functional music in neurological gait rehabilitation and its use in combination with treadmill training.	RCT<br><br>registration trial: DRKS00014603

8.	Bella et al. (2017)	idiopathic Parkinson’s disease	34	RAS	4 weeks	UPDRS; H&Y; ADL; CONSORT Statement; PEDro scale; Vicon MX Motion Capture System; BAASTA; Roland SPD-6 MIDI percussion pad	RAS-based training programme, customized according to the patient's preferred cadence, has improved follicular-temporal gait parameters, such as speed and step length, under uncued walking conditions. This effect was maintained 1 month after the end of the training programme.	FP7 Initial Training Network - grant agreement no. 238157

Abbreviations: **RAS** - Rhythmic Auditory Stimulation; **H&Y** - Hoehn and Yahr Scale; **MBEST -** Mini-BESTest; **N-FOGQ** - New Freezing of Gait Questionnaire**; MDS-UPDRS -** Movement Disorder Society-Unified Parkinson’s Disease Rating Scale; **UPDRS** - Unified Parkinson’s Disease Rating Scale; **BBS** - Berg Balance Scale; **9-HPT** - Nine-Hole Peg Test; **TUG** - Timed Up and Go test; **FMA** – Fugl-Meyer Assessment; **MMSE** - Mini Mental Status Examination; **FES-I** - Falls Efficacy Scale-International; **FES** - Falls Efficacy Scale; **FAB** - Frontal Assessment Battery; **FIM** - Functional Independence Measure; **SPPB** - Short Physical Performance Battery; **GDS** - Geriatric Depression Scale; **PDQ-8** - Parkinson’s Disease Quality of Life Questionnaire; **ABC** - Activities specific balance confidence; **FOGQ** - Freezing of Gait Questionnaire; **FGS** - Fast Gait Speed Test; **LOC** - a gait analysis with the locometre; **3MWT** - Three-Minute Walk Test; **IEB** - Instrumental Evaluation of Balance; **ADL** - Schwab and England Activities of Daily Living; **BAASTA** - Battery for the Assessment of Auditory Sensorimotor and Timing Abilities; **6MWT -** Six-Minute Walk Test.
