# Supplementary material for: Neurologic Music Therapy in Geriatric Rehabilitation: A Systematic Review
Source: Healthcare (Basel). 2022 Oct 31;10(11):2187. doi: 10.3390/healthcare10112187 (PMC9690210; doi:10.3390/healthcare10112187)
Supplement: Supplementary file 1 [file healthcare-10-02187-s001.zip › File S4_Exluded Fulltext.pdf]

Exluded Fulltext (N=79)

No.	Reference (author, year)	Motivation
1.	[1]	Application of music therapy, where no MT methods or techniques are specified.
2.	[2]	Age of participants from 47 to 82 years.
3.	[3]	Use of active music therapy.
4.	[4]	Use of individual MT sessions, no use of NMT techniques.
5.	[5]	Application of visual arts and reminiscence music.
6.	[6]	Application of receptive methods of music therapy.
7.	[7]	Application of music therapy, where no MT methods or techniques are specified.
8.	[8]	Application of musical interventions, i.e. listening to music, singing and playing instruments.
9.	[9]	Application of musical interventions combined with dance.
10.	[10]	Application of creative and receptive music therapy.
11.	[11]	Application of music-based intervention
12.	[12]	Application of music therapy, where no MT methods or techniques are specified.
13.	[13]	Application of Educational Music Therapy Interventions (song-based). Study design: a Cluster-Randomized Pilot Effectiveness Study.
14.	[14]	Application of music-based intervention.
15.	[15]	Application of rhythm-centred music making method.
16.	[16]	Study design: cross-sectional study
17.	[17]	Application of rhythm and music-based therapy. Age of participants from 50 to 75 years.
18.	[18]	Age of participants from 18 to 85 years and use of receptive music therapy.
19.	[19]	Study design: prospective randomized study and application of daily listening to experiential/traditional music.
20.	[20]	Age of participants from 41 to 62 years.
21.	[21]	Age of participants from 31 to 75 years.
22.	[22]	Participants: 16 healthy participants who reported a negative history of neurological and psychiatric problems; 20-22 year-old persons.

23.	[23]	Participants: adolescents with acquired brain injury.
24.	[24]	Study design: a single blind randomized controlled pre-/post-test; age of participants from 34 to 45 years.
25.	[25]	Application of active music therapy.
26.	[26]	Age of participants from 23 to 31 years, the use of active music therapy.
27.	[27]	Application of the Rhythm and Music Therapy (R-MT).
28.	[28]	Application of the Rhythm and Music Therapy (R-MT).
29.	[29]	Application of a single-session music intervention (SMI); comparative study.
30.	[30]	Application of music-supported therapy on a piano.
31.	[31]	Application of music therapy; healthy participants; mean age of 24 years.
32.	[32]	Study design: pilot study.
33.	[33]	Application of active music therapy.
34.	[34]	Application of receptive methods of music therapy.
35.	[35]	Application of receptive methods of music therapy; elderly surgery patients.
36.	[36]	Application of receptive methods of music therapy; elderly surgery patients.
37.	[37]	Application of receptive methods of music therapy.
38.	[38]	Healthy elderly women.
39.	[39]	Not RCT.
40.	[40]	Lack of information about the study design (not RCT); age of participants from 58 to 75 years.
41.	[41]	Application of dance therapy.
42.	[42]	Application of dance movement therapy.
43.	[43]	Participants: 28 healthy female (age of participants from 18 to 25 years).
44.	[44]	Application of music intervention before bedtime; age of participants from 50 to 75 years.

45.	[45]	Study design: A multi-center observational study; use of the “Music Care” application.
46.	[46]	Application of dance therapy.
47.	[47]	Application of dance therapy.
48.	[48]	Application of natural sounds, no NMT techniques.
49.	[49]	Study design: review.
50.	[50]	Application of music therapy, where no MT methods or techniques are specified; age of participants from 45 to 85 years.
51.	[51]	Application of music therapy as an Anti-Oppressive Practice; not strictly applicable to psychogeriatric patients.
52.	[52]	Age of participants from 14 to 60 years; Application of music therapy, where no MT methods or techniques are specified.
53.	[53]	Application of music therapy.
54.	[54]	Application of music therapy (active and receptive).
55.	[55]	Age of participants from 27 to 57 years (average age, 42 years); application of music therapy.
56.	[56]	Healthy participants.
57.	[57]	Study design: a controlled, non-randomized clinical trial; no use of NMT techniques.
58.	[58]	No use of NMT techniques in psychogeriatry.
59.	[59]	Participants: healthy young adults.
60.	[60]	Participants: healthy young adults.
61.	[61]	Application of music intervention (active).
62.	[62]	No use of NMT techniques in psychogeriatry.
63.	[63]	No use of NMT techniques in psychogeriatry.
64.	[64]	No strictly psychogeriatric disorders in patients.

65.	[65]	Application of music therapy; no use of NMT techniques.
66.	[66]	No use of NMT techniques; use of the created application based on rhythmic capabilities.
67.	[67]	Study design: cross-sectional study.
68.	[68]	No use of NMT techniques in psychogeriatry.
69.	[69]	The study is not RCT.
70.	[70]	The study is not RCT.
71.	[71]	The study is not RCT.
72.	[72]	Age of participants from 16 to 60 years.
73.	[73]	Age of participants from 33 to 76 years.
74.	[74]	The study is not RCT; Application of music therapy; no use of NMT techniques.
75.	[75]	Age of participants (mean age) 21.27 ± 2.74 years; Information about the design of the study being inspired by NMT techniques. The study is not RCT.
76.	[52]	The patients received a combination of music and auditory stimulation; no use of NMT techniques.
77.	[76]	No use of NMT techniques. Interventions for people with dementia in residential aged care: group music therapy (GMT) and recreational choir singing (RCS).
78.	[77]	Age of participants from 40 to 80 years. Insufficient information on interventions that do not allow a clear statement on the use of the NMT sensorimotor technique.
79.	[78]	The average age of the patients was 49.78 (SD = 15.55) years; no use of NMT techniques.

References:

1. Lyu, J.; Zhang, J.; Mu, H.; Li, W.; Champ, M.; Xiong, Q.; Gao, T.; Xie, L.; Jin, W.; Yang, W.; et al. The Effects of Music Therapy on Cognition, Psychiatric Symptoms, and Activities of Daily Living in Patients with Alzheimer's Disease. *J. Alzheimers Dis.* **2018**, *64*, 1347–1358, doi:10.3233/JAD-180183.
2. Haro-Martínez, A.M.; Lubrini, G.; Madero-Jarabo, R.; Díez-Tejedor, E.; Fuentes, B. Melodic Intonation Therapy in Post-Stroke Nonfluent Aphasia: A Randomized Pilot Trial. *Clin. Rehabil.* **2019**, *33*, 44–53, doi:10.1177/0269215518791004.
3. Giovagnoli, A.R.; Manfredi, V.; Schifano, L.; Paterlini, C.; Parente, A.; Tagliavini, F. Combining Drug and Music Therapy in Patients with Moderate Alzheimer's Disease: A

Randomized Study. *Neurol. Sci.* **2018**, *39*, 1021–1028, doi:10.1007/s10072-018-3316-3.
4. Sánchez, A.; Maseda, A.; Marante-Moar, M.P.; de Labra, C.; Lorenzo-López, L.; Millán-Calenti, J.C. Comparing the Effects of Multisensory Stimulation and Individualized Music Sessions on Elderly People with Severe Dementia: A Randomized Controlled Trial. *J. Alzheimers Dis.* **2016**, *52*, 303–315, doi:10.3233/JAD-151150.
5. Mahendran, R.; Gandhi, M.; Moorakonda, R.B.; Wong, J.; Kanchi, M.M.; Fam, J.; Rawtaer, I.; Kumar, A.P.; Feng, L.; Kua, E.H. Art Therapy Is Associated with Sustained Improvement in Cognitive Function in the Elderly with Mild Neurocognitive Disorder: Findings from a Pilot Randomized Controlled Trial for Art Therapy and Music Reminiscence Activity versus Usual Care. *Trials* **2018**, *19*, 615, doi:10.1186/s13063-018-2988-6.
6. Gök Ugur, H.; Yaman Aktaş, Y.; Orak, O.S.; Saglambilen, O.; Aydin Avci, İ. The Effect of Music Therapy on Depression and Physiological Parameters in Elderly People Living in a Turkish Nursing Home: A Randomized-Controlled Trial. *Aging Ment. Health* **2017**, *21*, 1280–1286, doi:10.1080/13607863.2016.1222348.
7. Han, J.W.; Lee, H.; Hong, J.W.; Kim, K.; Kim, T.; Byun, H.J.; Ko, J.W.; Youn, J.C.; Ryu, S.-H.; Lee, N.-J.; et al. Multimodal Cognitive Enhancement Therapy for Patients with Mild Cognitive Impairment and Mild Dementia: A Multi- Center, Randomized, Controlled, Double-Blind, Crossover Trial. *J. Alzheimers Dis.* **2016**, *55*, 787–796, doi:10.3233/JAD-160619.
8. Pitkänen, A.; Alanen, H.-M.; Kampman, O.; Suontaka-Jamalainen, K.; Leinonen, E. Implementing Physical Exercise and Music Interventions for Patients Suffering from Dementia on an Acute Psychogeriatric Inpatient Ward. *Nord. J. Psychiatry* **2019**, *73*, 401–408, doi:10.1080/08039488.2019.1645205.
9. Doi, T.; Verghese, J.; Makizako, H.; Tsutsumimoto, K.; Hotta, R.; Nakakubo, S.; Suzuki, T.; Shimada, H. Effects of Cognitive Leisure Activity on Cognition in Mild Cognitive Impairment: Results of a Randomized Controlled Trial. *J. Am. Med. Dir. Assoc.* **2017**, *18*, 686–691, doi:10.1016/j.jamda.2017.02.013.
10. Feng, K.; Shen, C.-Y.; Ma, X.-Y.; Chen, G.-F.; Zhang, M.-L.; Xu, B.; Liu, X.-M.; Sun, J.-J.; Zhang, X.-Q.; Liu, P.-Z.; et al. Effects of Music Therapy on Major Depressive Disorder: A Study of Prefrontal Hemodynamic Functions Using FNIRS. *Psychiatry Res.* **2019**, *275*, 86–93, doi:10.1016/j.psychres.2019.03.015.
11. Särkämö, T.; Laitinen, S.; Numminen, A.; Kurki, M.; Johnson, J.K.; Rantanen, P. Clinical and Demographic Factors Associated with the Cognitive and Emotional Efficacy of Regular Musical Activities in Dementia. *J. Alzheimers Dis.* **2015**, *49*, 767–781, doi:10.3233/JAD-150453.
12. Shirani Bidabadi, S.; Mehryar, A. Music Therapy as an Adjunct to Standard Treatment for Obsessive Compulsive Disorder and Co-Morbid Anxiety and Depression: A Randomized Clinical Trial. *J. Affect. Disord.* **2015**, *184*, 13–17, doi:10.1016/j.jad.2015.04.011.
13. Silverman, M.J. Comparing Educational Music Therapy Interventions via Stages of Recovery with Adults in an Acute Care Mental Health Setting: A Cluster-Randomized Pilot Effectiveness Study. *Community Ment. Health J.* **2019**, *55*, 624–630, doi:10.1007/s10597-019-00380-1.
14. Ho, R.T.; Fong, T.C.; Sing, C.; Lee, P.H.; Leung, A.B.; Chung, K.S.; Kwok, J.K. Managing Behavioral and Psychological Symptoms in Chinese Elderly with Dementia via Group-Based Music Intervention: A Cluster Randomized Controlled Trial. *Dementia* **2019**, *18*, 2785–2798, doi:10.1177/1471301218760023.
15. Yap, A.F.; Kwan, Y.H.; Tan, C.S.; Ibrahim, S.; Ang, S.B. Rhythm-Centred Music Making in Community Living Elderly: A Randomized Pilot Study. *BMC Complement. Altern. Med.* **2017**, *17*, 311, doi:10.1186/s12906-017-1825-x.
16. Marmelat, V.; Duncan, A.; Meltz, S.; Meidinger, R.L.; Hellman, A.M. Fractal Auditory Stimulation Has Greater Benefit for People with Parkinson's Disease Showing More

Random Gait Pattern. *Gait Posture* **2020**, *80*, 234–239, doi:10.1016/j.gaitpost.2020.05.021.
17. Bunketorp-Käll, L.; Pekna, M.; Pekny, M.; Blomstrand, C.; Nilsson, M. Effects of Horse-Riding Therapy and Rhythm and Music-Based Therapy on Functional Mobility in Late Phase after Stroke. *NeuroRehabilitation* **2019**, *45*, 483–492, doi:10.3233/NRE-192905.
18. Lee, C.-H.; Lai, C.-L.; Sung, Y.-H.; Lai, M.Y.; Lin, C.-Y.; Lin, L.-Y. Comparing Effects between Music Intervention and Aromatherapy on Anxiety of Patients Undergoing Mechanical Ventilation in the Intensive Care Unit: A Randomized Controlled Trial. *Qual. Life Res. Int. J. Qual. Life Asp. Treat. Care Rehabil.* **2017**, *26*, 1819–1829, doi:10.1007/s11136-017-1525-5.
19. Fotakopoulos, G.; Kotlia, P. The Value of Exercise Rehabilitation Program Accompanied by Experiential Music for Recovery of Cognitive and Motor Skills in Stroke Patients. *J. Stroke Cerebrovasc. Dis. Off. J. Natl. Stroke Assoc.* **2018**, *27*, 2932–2939, doi:10.1016/j.jstrokecerebrovasdis.2018.06.025.
20. Impellizzeri, F.; Leonardi, S.; Latella, D.; Maggio, M.G.; Foti Cuzzola, M.; Russo, M.; Sessa, E.; Bramanti, P.; De Luca, R.; Calabrò, R.S. An Integrative Cognitive Rehabilitation Using Neurologic Music Therapy in Multiple Sclerosis: A Pilot Study. *Medicine (Baltimore)* **2020**, *99*, e18866, doi:10.1097/MD.0000000000018866.
21. Street, A.J.; Magee, W.L.; Bateman, A.; Parker, M.; Odell-Miller, H.; Fachner, J. Home-Based Neurologic Music Therapy for Arm Hemiparesis Following Stroke: Results from a Pilot, Feasibility Randomized Controlled Trial. *Clin. Rehabil.* **2018**, *32*, 18–28, doi:10.1177/0269215517717060.
22. Cheung, M.-C.; Chan, A.S.; Liu, Y.; Law, D.; Wong, C.W.Y. Music Training Is Associated with Cortical Synchronization Reflected in EEG Coherence during Verbal Memory Encoding. *PloS One* **2017**, *12*, e0174906, doi:10.1371/journal.pone.0174906.
23. Kim, S.J.; Shin, Y.-K.; Yoo, G.E.; Chong, H.J.; Cho, S.-R. Changes in Gait Patterns Induced by Rhythmic Auditory Stimulation for Adolescents with Acquired Brain Injury. *Ann. N. Y. Acad. Sci.* **2016**, *1385*, 53–62, doi:10.1111/nyas.13294.
24. van Alphen, R.; Stams, G.J.J.M.; Hakvoort, L. Musical Attention Control Training for Psychotic Psychiatric Patients: An Experimental Pilot Study in a Forensic Psychiatric Hospital. *Front. Neurosci.* **2019**, *13*, 570, doi:10.3389/fnins.2019.00570.
25. Raglio, A.; Oasi, O.; Gianotti, M.; Rossi, A.; Goulene, K.; Stramba-Badiale, M. Improvement of Spontaneous Language in Stroke Patients with Chronic Aphasia Treated with Music Therapy: A Randomized Controlled Trial. *Int. J. Neurosci.* **2016**, *126*, 235–242, doi:10.3109/00207454.2015.1010647.
26. Baur, K.; Speth, F.; Nagle, A.; Riener, R.; Klamroth-Marganska, V. Music Meets Robotics: A Prospective Randomized Study on Motivation during Robot Aided Therapy. *J. Neuroengineering Rehabil.* **2018**, *15*, 79, doi:10.1186/s12984-018-0413-8.
27. Pohl, P.; Carlsson, G.; Bunketorp Käll, L.; Nilsson, M.; Blomstrand, C. Experiences from a Multimodal Rhythm and Music-Based Rehabilitation Program in Late Phase of Stroke Recovery - A Qualitative Study. *PloS One* **2018**, *13*, e0204215, doi:10.1371/journal.pone.0204215.
28. Bunketorp-Käll, L.; Lundgren-Nilsson, Å.; Samuelsson, H.; Pekny, T.; Blomvé, K.; Pekna, M.; Pekny, M.; Blomstrand, C.; Nilsson, M. Long-Term Improvements After Multimodal Rehabilitation in Late Phase After Stroke: A Randomized Controlled Trial. *Stroke* **2017**, *48*, 1916–1924, doi:10.1161/STROKEAHA.116.016433.
29. Chen, S.-C.; Yeh, M.-L.; Chang, H.-J.; Lin, M.-F. Music, Heart Rate Variability, and Symptom Clusters: A Comparative Study. *Support. Care Cancer Off. J. Multinatl. Assoc. Support. Care Cancer* **2020**, *28*, 351–360, doi:10.1007/s00520-019-04817-x.
30. van Vugt, F.T.; Kafczyk, T.; Kuhn, W.; Rollnik, J.D.; Tillmann, B.; Altenmüller, E. The Role of Auditory Feedback in Music-Supported Stroke Rehabilitation: A Single-Blinded Randomised Controlled Intervention. *Restor. Neurol. Neurosci.* **2016**, *34*, 297–311, doi:10.3233/RNN-150588.
31. Santhosh, A.K.; Sangilirajan, M.; Nizar, N.; Radhamani, R.; Kumar, D.; Bodda, S.; Diwakar, S. Computational Exploration of Neural Dynamics Underlying Music Cues

Among Trained and Amateur Subjects. *Procedia Comput. Sci.* **2020**, *171*, 1839–1847, doi:10.1016/j.procs.2020.04.197.
32. Bukowska, A.A.; Krężałek, P.; Mirek, E.; Bujas, P.; Marchewka, A. Neurologic Music Therapy Training for Mobility and Stability Rehabilitation with Parkinson's Disease – A Pilot Study. *Front. Hum. Neurosci.* **2016**, *9*, doi:10.3389/fnhum.2015.00710.
33. Gómez Gallego, M.; Gómez García, J. Music Therapy and Alzheimer's Disease: Cognitive, Psychological, and Behavioural Effects. *Neurol. Engl. Ed.* **2017**, *32*, 300–308, doi:10.1016/j.nrleng.2015.12.001.
34. Burrai, F.; Sanna, G.D.; Moccia, E.; Morlando, F.; Cosentino, E.R.; Bui, V.; Micheluzzi, V.; Borghi, C.; Parodi, G. Beneficial Effects of Listening to Classical Music in Patients With Heart Failure: A Randomized Controlled Trial. *J. Card. Fail.* **2020**, *26*, 541–549, doi:10.1016/j.cardfail.2019.12.005.
35. Çetinkaya, F. Effect of Listening to Music on Postoperative Cognitive Function in Older Adults After Hip or Knee Surgery: A Randomized Controlled Trial. *J. Perianesth. Nurs.* **2019**, *34*, 919–928, doi:10.1016/j.jopan.2019.03.001.
36. Kavakli, A.S.; Kavrut Ozturk, N.; Yavuzel Adas, H.; Kudsioglu, S.T.; Ayoglu, R.U.; Özmen, S.; Sagdic, K.; Yapici, N. The Effects of Music on Anxiety and Pain in Patients during Carotid Endarterectomy under Regional Anesthesia: A Randomized Controlled Trial. *Complement. Ther. Med.* **2019**, *44*, 94–101, doi:10.1016/j.ctim.2019.04.005.
37. Tanaka, Y.; Nogawa, H. Evaluating the Effects of Singing Songs in Ethnic Music Therapy for Dementia Patients with a Novel Near-Infrared Spectroscopy (Data Analysis Method). *Int. J. Gerontol.* **2015**, *9*, 7–14, doi:10.1016/j.ijge.2014.01.006.
38. Moon, S.; Park, J.; Yang, S. The Effects of Therapeutic Singing on Vocal Functions of the Elderly: A Study on Korean Elderly. *J. Voice* **2020**, doi:10.1016/j.jvoice.2020.06.007.
39. Cho, H.; Kim, K. Effects of Action Observation Training with Auditory Stimulation on Static and Dynamic Balance in Chronic Stroke Patients. *J. Stroke Cerebrovasc. Dis.* **2020**, *29*, 104775, doi:10.1016/j.jstrokecerebrovasdis.2020.104775.
40. Song, J.-H.; Zhou, P.-Y.; Cao, Z.-H.; Ding, Z.-G.; Chen, H.-X.; Zhang, G.-B. Rhythmic Auditory Stimulation with Visual Stimuli on Motor and Balance Function of Patients with Parkinson's Disease. *Eur. Rev. Med. Pharmacol. Sci.* **2015**, *19*, 2001–2007.
41. Rios Romenets, S.; Anang, J.; Fereshtehnejad, S.-M.; Pelletier, A.; Postuma, R. Tango for Treatment of Motor and Non-Motor Manifestations in Parkinson's Disease: A Randomized Control Study. *Complement. Ther. Med.* **2015**, *23*, 175–184, doi:10.1016/j.ctim.2015.01.015.
42. Esmail, A.; Vrinceanu, T.; Lussier, M.; Predovan, D.; Berryman, N.; Houle, J.; Karelis, A.; Grenier, S.; Minh Vu, T.T.; Villalpando, J.M.; et al. Effects of Dance/Movement Training vs. Aerobic Exercise Training on Cognition, Physical Fitness and Quality of Life in Older Adults: A Randomized Controlled Trial. *J. Bodyw. Mov. Ther.* **2020**, *24*, 212–220, doi:10.1016/j.jbmt.2019.05.004.
43. do Amaral, J.A.T.; Guida, H.L.; de Abreu, L.C.; Barnabé, V.; Vanderlei, F.M.; Valenti, V.E. Effects of Auditory Stimulation with Music of Different Intensities on Heart Period. *J. Tradit. Complement. Med.* **2016**, *6*, 23–28, doi:10.1016/j.jtcme.2014.11.032.
44. Huang, C.-Y.; Chang, E.-T.; Lai, H.-L. Comparing the Effects of Music and Exercise with Music for Older Adults with Insomnia. *Appl. Nurs. Res.* **2016**, *32*, 104–110, doi:10.1016/j.apnr.2016.06.009.
45. Guétin, S.; Diego, E. de; Mohy, F.; Adolphe, C.; Hoareau, G.; Touchon, J.; Thayer, J.F.; Koenig, J. A Patient-Controlled, Smartphone-Based Music Intervention to Reduce Pain—A Multi-Center Observational Study of Patients with Chronic Pain. *Eur. J. Integr. Med.* **2016**, *8*, 182–187, doi:10.1016/j.eujim.2016.01.002.
46. Shanahan, J.; Morris, M.E.; Bhriain, O.N.; Volpe, D.; Lynch, T.; Clifford, A.M. Dancing for Parkinson Disease: A Randomized Trial of Irish Set Dancing Compared With Usual Care. *Arch. Phys. Med. Rehabil.* **2017**, *98*, 1744–1751, doi:10.1016/j.apmr.2017.02.017.
47. Van Geel, F.; Van Asch, P.; Veldkamp, R.; Feys, P. Effects of a 10-Week Multimodal Dance and Art Intervention Program Leading to a Public Performance in Persons with

Multiple Sclerosis - A Controlled Pilot-Trial. *Mult. Scler. Relat. Disord.* **2020**, *44*, 102256, doi:10.1016/j.msard.2020.102256.
48. Saadatmand, V.; Rejeh, N.; Heravi-Karimooi, M.; Tadrisi, S.D.; Vaismoradi, M.; Jordan, S. Effects of Natural Sounds on Pain: A Randomized Controlled Trial with Patients Receiving Mechanical Ventilation Support. *Pain Manag. Nurs.* **2015**, *16*, 483–492, doi:10.1016/j.pmn.2014.09.006.
49. Shi, E.R.; Zhang, Q. A Domain-General Perspective on the Role of the Basal Ganglia in Language and Music: Benefits of Music Therapy for the Treatment of Aphasia. *Brain Lang.* **2020**, *206*, 104811, doi:10.1016/j.bandl.2020.104811.
50. Facai, L.; Dehong, H.; Nana, H.; Yihuang, G.; Yunchuan, W. Effect of Music Therapy Derived from the Five Elements in Traditional Chinese Medicine on Post-Stroke Depression. *J. Tradit. Chin. Med.* **2017**, *37*, 675–680, doi:10.1016/S0254-6272(17)30322-9.
51. Baines, S.; Edwards, J. A Constructivist Grounded Theory Research Project Studying Music Therapy as an Anti-Oppressive Practice in Long-Term and Psychiatric Residential Care. *Arts Psychother.* **2018**, *60*, 1–8, doi:10.1016/j.aip.2018.04.003.
52. Froutan, R.; Eghbali, M.; Hoseini, S.H.; Mazloom, S.R.; Yekaninejad, M.S.; Boostani, R. The Effect of Music Therapy on Physiological Parameters of Patients with Traumatic Brain Injury: A Triple-Blind Randomized Controlled Clinical Trial. *Complement. Ther. Clin. Pract.* **2020**, *40*, 101216, doi:10.1016/j.ctcp.2020.101216.
53. Deatrich, K.G.; Prout, M.F.; Boyer, B.A.; Yoder, S.E. Effectiveness of Group Music Therapy in a Psychiatric Hospital: A Randomized Pilot Study of Treatment Outcome. *Int. J. Group Psychother.* **2016**, *66*, 592–617, doi:10.1080/00207284.2016.1190239.
54. Warth, M.; Kessler, J.; Koenig, J.; Wormit, A.F.; Hillecke, T.K.; Bardenheuer, H.J. Music Therapy to Promote Psychological and Physiological Relaxation in Palliative Care Patients: Protocol of a Randomized Controlled Trial. *BMC Palliat. Care* **2014**, *13*, 60, doi:10.1186/1472-684X-13-60.
55. Degli Stefani, M.; Biasutti, M. Effects of Music Therapy on Drug Therapy of Adult Psychiatric Outpatients: A Pilot Randomized Controlled Study. *Front. Psychol.* **2016**, *7*, doi:10.3389/fpsyg.2016.01518.
56. Imtiaz, D.; Anwar, Y.; Khan, A. Wearable Sensors and a Multisensory Music and Reminiscence Therapies Application: To Help Reduce Behavioral and Psychological Symptoms in Person with Dementia. *Smart Health* **2020**, 100140, doi:10.1016/j.smhl.2020.100140.
57. Luvizutto, G.J.; Katlen da Silva, L.; Silva Brito, T.S.; Pascucci Sande de Souza, L.A. Music-Based Physical Therapy in Parkinson's Disease: An Approach Based on International Classification of Functioning, Disability and Health. *J. Bodyw. Mov. Ther.* **2020**, doi:10.1016/j.jbmt.2020.08.015.
58. Huang, C.-Y.; Chang, E.-T.; Hsieh, Y.-M.; Lai, H.-L. Effects of Music and Music Video Interventions on Sleep Quality: A Randomized Controlled Trial in Adults with Sleep Disturbances. *Complement. Ther. Med.* **2017**, *34*, 116–122, doi:10.1016/j.ctim.2017.08.015.
59. Ready, E.A.; McGarry, L.M.; Rinchon, C.; Holmes, J.D.; Grahn, J.A. Beat Perception Ability and Instructions to Synchronize Influence Gait When Walking to Music-Based Auditory Cues. *Gait Posture* **2019**, *68*, 555–561, doi:10.1016/j.gaitpost.2018.12.038.
60. Kasdan, A.; Kiran, S. Please Don't Stop the Music: Song Completion in Patients with Aphasia. *J. Commun. Disord.* **2018**, *75*, 72–86, doi:10.1016/j.jcomdis.2018.06.005.
61. Tang, Q.; Zhou, Y.; Yang, S.; Thomas, W.K.S.; Smith, G.D.; Yang, Z.; Yuan, L.; Chung, J.W. Effect of Music Intervention on Apathy in Nursing Home Residents with Dementia. *Geriatr. Nur. (Lond.)* **2018**, *39*, 471–476, doi:10.1016/j.gerinurse.2018.02.003.
62. Ji, L.; Bai, J.-J.; Sun, J.; Ming, Y.; Chen, L.-R. Effect of Combining Music Media Therapy with Lower Extremity Exercise on Elderly Patients with Diabetes Mellitus. *Int. J. Nurs. Sci.* **2015**, *2*, 243–247, doi:10.1016/j.ijnss.2015.07.008.
63. Stegemöller, E.L.; Izbicki, P.; Hibbing, P. The Influence of Moving with Music on Motor Cortical Activity. *Neurosci. Lett.* **2018**, *683*, 27–32, doi:10.1016/j.neulet.2018.06.030.

64. Argstatter, H.; Grapp, M.; Hutter, E.; Plinkert, P.K.; Bolay, H.-V. The Effectiveness of Neuro-Music Therapy According to the Heidelberg Model Compared to a Single Session of Educational Counseling as Treatment for Tinnitus: A Controlled Trial. *J. Psychosom. Res.* **2015**, *78*, 285–292, doi:10.1016/j.jpsychores.2014.08.012.
65. Espí-López, G.V.; Inglés, M.; Ruescas-Nicolau, M.-A.; Moreno-Segura, N. Effect of Low-Impact Aerobic Exercise Combined with Music Therapy on Patients with Fibromyalgia. A Pilot Study. *Complement. Ther. Med.* **2016**, *28*, 1–7, doi:10.1016/j.ctim.2016.07.003.
66. Dauvergne, C.; Bégel, V.; Gény, C.; Puyjarinet, F.; Laffont, I.; Dalla Bella, S. Home-Based Training of Rhythmic Skills with a Serious Game in Parkinson's Disease: Usability and Acceptability. *Ann. Phys. Rehabil. Med.* **2018**, *61*, 380–385, doi:10.1016/j.rehab.2018.08.002.
67. Horin, A.P.; Harrison, E.C.; Rawson, K.S.; Earhart, G.M. Finger Tapping as a Proxy for Gait: Similar Effects on Movement Variability during External and Self-Generated Cueing in People with Parkinson's Disease and Healthy Older Adults. *Ann. Phys. Rehabil. Med.* **2020**, doi:10.1016/j.rehab.2020.05.009.
68. Gomes, R.L.; Vanderlei, L.C.M.; Garner, D.M.; de Marco, R.L.; Gonzaga, L.A.; Valenti, V.E. The Effects of Musical Auditory Stimulation on Cardiorespiratory Variables after Aerobic Exercise. *Sci. Sports* **2018**, *33*, 221–227, doi:10.1016/j.scispo.2018.01.002.
69. Pau, M.; Corona, F.; Pili, R.; Casula, C.; Sors, F.; Agostini, T.; Cossu, G.; Guicciardi, M.; Murgia, M. Effects of Physical Rehabilitation Integrated with Rhythmic Auditory Stimulation on Spatio-Temporal and Kinematic Parameters of Gait in Parkinson's Disease. *Front. Neurol.* **2016**, *7*, doi:10.3389/fneur.2016.00126.
70. Kadivar, Z.; Corcos, D.M.; Foto, J.; Hondzinski, J.M. Effect of Step Training and Rhythmic Auditory Stimulation on Functional Performance in Parkinson Patients. *Neurorehabil. Neural Repair* **2011**, *25*, 626–635, doi:10.1177/1545968311401627.
71. Erra, C.; Mileti, I.; Germanotta, M.; Petracca, M.; Imbimbo, I.; Biase, A.; Rossi, S.; Ricciardi, D.; Pacilli, A.; Di SIpio, E.; et al. Immediate Effects of Rhythmic Auditory Stimulation on Gait Kinematics in Parkinson's Disease ON/OFF Medication. *Clin. Neurophysiol.* **2019**, *130*, doi:10.1016/j.clinph.2019.07.013.
72. Martínez-Molina, N.; Siponkoski, S.-T.; Kuusela, L.; Laitinen, S.; Holma, M.; Ahlfors, M.; Jordan-Kilkki, P.; Ala-Kauhaluoma, K.; Melkas, S.; Pekkola, J.; et al. Resting-State Network Plasticity Induced by Music Therapy after Traumatic Brain Injury. *Neural Plast.* **2021**, *2021*, 1–18, doi:10.1155/2021/6682471.
73. Haire, C.M.; Vuong, V.; Tremblay, L.; Patterson, K.K.; Chen, J.L.; Thaut, M.H. Effects of Therapeutic Instrumental Music Performance and Motor Imagery on Chronic Post-Stroke Cognition and Affect: A Randomized Controlled Trial. *NeuroRehabilitation* **2021**, *48*, 195–208, doi:10.3233/NRE-208014.
74. Geipel, J.; Koenig, J.; Hillecke, T.K.; Resch, F. Short-Term Music Therapy Treatment for Adolescents with Depression – A Pilot Study. *Arts Psychother.* **2022**, *77*, 101874, doi:10.1016/j.aip.2021.101874.
75. Curzel, F.; Brigadoi, S.; Cutini, S. FNIRS & E-Drum: An Ecological Approach to Monitor Hemodynamic and Behavioural Effects of Rhythmic Auditory Cueing Training. *Brain Cogn.* **2021**, *151*, 105753, doi:10.1016/j.bandc.2021.105753.
76. Baker, F.A.; Lee, Y.-E.C.; Sousa, T.V.; Stretton-Smith, P.A.; Tamplin, J.; Sveinsdottir, V.; Geretsegger, M.; Wake, J.D.; Assmus, J.; Gold, C. Clinical Effectiveness of Music Interventions for Dementia and Depression in Elderly Care (MIDDEL): Australian Cohort of an International Pragmatic Cluster-Randomised Controlled Trial. *Lancet Healthy Longev.* **2022**, *3*, e153–e165, doi:10.1016/S2666-7568(22)00027-7.
77. Wang, Y.; Pan, W.-Y.; Li, F.; Ge, J.-S.; Zhang, X.; Luo, X.; Wang, Y.-L. Effect of Rhythm of Music Therapy on Gait in Patients with Stroke. *J. Stroke Cerebrovasc. Dis.* **2021**, *30*, 105544, doi:10.1016/j.jstrokecerebrovasdis.2020.105544.
78. Kang, S.; Shin, J.-H.; Kim, I.Y.; Lee, J.; Lee, J.-Y.; Jeong, E. Patterns of Enhancement in Paretic Shoulder Kinematics after Stroke with Musical Cueing. *Sci. Rep.* **2020**, *10*, 18109, doi:10.1038/s41598-020-75143-0.
