# Supplementary material for: Neurologic Music Therapy in Geriatric Rehabilitation: A Systematic Review
Source: Healthcare (Basel). 2022 Oct 31;10(11):2187. doi: 10.3390/healthcare10112187 (PMC9690210; doi:10.3390/healthcare10112187)
Supplement: Supplementary file 1 [file healthcare-10-02187-s001.zip › [File S1_PICO].pdf]

Title: **Neurologic Music Therapy in Geriatric Rehabilitation: A Systematic Review.**

Objective: feasibility and effectiveness of NMT techniques in geriatric patients

Patients: patients aged 60 years and over with neurodegenerative diseases

Interventions: use of NMT techniques

Comparison: no-NMT and all different interventions

Outcome: improvement in psychogeriatric disorders

Key words

**POPULATION**		**INTERVENTION**		**OUTCOME**
“Psychogeriatric disorders”		“Neurologic Music Therapy”		"Neuropsychological Tests"[Mesh]
“Mental disorders”		“Rhythmic Auditory Stimulation – RAS”		"Mental Status and Dementia Tests "[Mesh]
“Dementia”		“Patterned Sensory Enhancement – PSE”		“Mini Mental State Examination”
“Parkinson’s Disease”		“Therapeutic Instrumental Music Performance – TIMP”		“Mini Mental Status Examination”
“Delirium”		“Melodic Intonation Therapy – MIT”		“The Psychogeriatric Assessment Scales (PAS)”
“Depression”		“Musical Speech Stimulation – MUSTIM”		“Functional Gait Assessment”
“Psychotic disorders”		“Rhythmic Speech Cueing – RSC”		“Tinetti Falls Efficacy Scale”
“Anxiety disorders”		“Vocal Intonation Therapy – VIT”		“Berg’s Balance Scale”
“Neurodegenerative diseases”		“Therapeutic Singing – TS"		“Timed Up and Go test”
“Somatic disease”		“Oral Motor and Respiratory Exercises – OMREX”		“Functional Independence Measure”
“Elderly”		“Music Sensory Orientation Training – MSOT”		“Geriatric Depression Scale”
“Cardiovascular disease”		“Music Neglect Training – MNT”		“Schwab and England Activities of Daily Living”
“Neurocognitive Disorders”		“Auditory Perception Training – APT”		“Battery for the Assessment of Auditory Sensorimotor and Timing Abilities”
“Schizophrenia”		“Musical Attention Control Training – MACT”		“The World Health Organization Quality of Life (WHOQOL)”

“Bipolar Disorder”		“Musical Mnemonic Training – MMT”		“Biomechanical measurement”
		“Associative Mood and Memory Training – AMMT”		
		“Musical Executive Functions Training – MEFT”		
		“Music Psychotherapy and Counseling – MPC”		

PubMED, ScienceDirect and EBSCOhost

1. "Psychogeriatric disorders"[All Fields] OR "Mental disorders"[All Fields] OR "Dementia"[All Fields] OR "Parkinson's Disease"[All Fields] OR "Delirium"[All Fields] OR "Depression"[All Fields] OR "Psychotic disorders"[All Fields] OR "Anxiety disorders"[All Fields] OR "Neurodegenerative diseases"[All Fields] OR "Somatic disease"[All Fields] OR "Elderly"[All Fields] OR "Multiple Sclerosis"[All Fields] OR "Stroke"[All Fields] OR "Traumatic Brain Injury"[All Fields] OR "Neurocognitive Disorders"[All Fields] OR "Schizophrenia"[All Fields] OR "Bipolar disorder"[All Fields]

2. ((((((((((((((((((((((((((((((((("Neurologic Music Therapy") OR (NMT)) OR ("Rhythmic Auditory Stimulation")) OR (RAS)) OR ("Patterned Sensory Enhancement")) OR (PSE)) OR ("Therapeutic Instrumental Music Performance")) OR (TIMP)) OR ("Melodic Intonation Therapy")) OR (MIT)) OR ("Musical Speech Stimulation")) OR (MUSTIM)) OR ("Rhythmic Speech Cueing")) OR (RSC)) OR ("Vocal Intonation Therapy")) OR (VIT)) OR ("Therapeutic Singing")) OR (TS)) OR ("Oral Motor and Respiratory Exercises")) OR (OMREX)) OR ("Music Sensory Orientation Training")) OR (MSOT)) OR ("Music Neglect Training")) OR (MNT)) OR ("Auditory Perception Training")) OR (APT)) OR ("Musical Attention Control Training")) OR (MACT)) OR ("Musical Mnemonic Training")) OR (MMT)) OR ("Associative Mood and Memory Training")) OR (AMMT)) OR ("Musical Executive Functions Training")) OR ("Music Psychotherapy and Counseling")) OR (MPC)

3. ((((((((((((("Neuropsychological Tests"[Mesh]) OR ("Mental Status and Dementia Tests "[Mesh])) OR ("Mini Mental State Examination")) OR ("Mini Mental Status Examination")) OR ("The Psychogeriatric Assessment Scales (PAS)")) OR ("Functional Gait Assessment")) OR ("Tinetti Falls Efficacy Scale")) OR ("Berg’s Balance Scale")) OR ("Timed Up and Go test")) OR ("Functional Independence Measure")) OR ("Geriatric Depression Scale")) OR ("Schwab and England Activities of Daily Living")) OR ("Battery for the Assessment of Auditory Sensorimotor and Timing Abilities")) OR ("The World Health Organization Quality of Life (WHOQOL)")) OR ("Biomechanical measurement")
4. (#1 AND #2 AND #3)
