# Supplementary material for: Neurologic Music Therapy in Geriatric Rehabilitation: A Systematic Review
Source: Healthcare (Basel). 2022 Oct 31;10(11):2187. doi: 10.3390/healthcare10112187 (PMC9690210; doi:10.3390/healthcare10112187)
Supplement: Supplementary file 1 [file healthcare-10-02187-s001.zip › [File S2_Risk of Bias].pdf]

| | | | | | |
|---|---|---|---|---|---|
| **Unique ID** | Capato et.al. 2020 | **Study ID** | | **Assessor** | |
| **Ref or Label** | | **Aim** | assignment to intervention (the 'intention-to-treat' effect) | | |
| **Experimental** | | **Comparator** | | **Source** | Journal article(s) |
| **Outcome** | | **Results** | | **Weight** | 1 |

| Domain | Signalling question | Response | Comments |
|---|---|---|---|
| **Bias arising from the randomization process** | 1.1 Was the allocation sequence random? | Y | Use a computer random number generator.<br>Use of opaque sealed envelopes. |
| | 1.2 Was the allocation sequence concealed until participants were enrolled and assigned to interventions? | Y | |
| | 1.3 Did baseline differences between intervention groups suggest a problem with the randomization process? | PN | |
| | **Risk of bias judgement** | **Low** | |
| **Bias due to deviations from intended interventions** | 2.1.Were participants aware of their assigned intervention during the trial? | N | Evaluators blinded to group designation. |
| | 2.2.Were carers and people delivering the interventions aware of participants' assigned intervention during the trial? | PN | |
| | 2.3. If Y/PY/NI to 2.1 or 2.2: Were there deviations from the intended intervention that arose because of the experimental context? | NA | |
| | 2.4 If Y/PY to 2.3: Were these deviations likely to have affected the outcome? | NA | |
| | 2.5. If Y/PY/NI to 2.4: Were these deviations from intended intervention balanced between groups? | NA | |
| | 2.6 Was an appropriate analysis used to estimate the effect of assignment to intervention? | Y | |
| | 2.7 If N/PN/NI to 2.6: Was there potential for a substantial impact (on the result) of the failure to analyse participants in the group to which they were randomized? | NA | |
| | **Risk of bias judgement** | **Low** | |
| **Bias due to missing outcome data** | 3.1 Were data for this outcome available for all, or nearly all, participants randomized? | N | Use of opaque sealed envelopes<br>Evaluators blinded to group designation<br>7 participants out of 25 dropped out, reasons |
| | 3.2 If N/PN/NI to 3.1: Is there evidence that result was not biased by missing outcome data? | PN | |
| | 3.3 If N/PN to 3.2: Could missingness in the outcome depend on its true value? | PN | |
| | 3.4 If Y/PY/NI to 3.3: Is it likely that missingness in the outcome depended on its true value? | NA | |
| | **Risk of bias judgement** | **Some concerns** | |
| **Bias in measurement of the outcome** | 4.1 Was the method of measuring the outcome inappropriate? | N | |
| | 4.2 Could measurement or ascertainment of the outcome have differed between intervention groups? | PN | |
| | 4.3 Were outcome assessors aware of the intervention received by study participants? | PN | |
| | 4.4 If Y/PY/NI to 4.3: Could assessment of the outcome have been influenced by knowledge of intervention received? | NA | |
| | 4.5 If Y/PY/NI to 4.4: Is it likely that assessment of the outcome was influenced by knowledge of intervention received? | NA | |
| | **Risk of bias judgement** | **Low** | |
| **Bias in selection of the reported result** | 5.1 Were the data that produced this result analysed in accordance with a pre-specified analysis plan that was finalized before unblinded outcome data were available for analysis? | PY | Evaluators blinded to group designation<br>All pre-specified outcomes reported. |
| | 5.2 ... multiple eligible outcome measurements (e.g. scales, definitions, time points) within the outcome domain? | PN | |
| | 5.3 ... multiple eligible analyses of the data? | PN | |
| | **Risk of bias judgement** | **Low** | |
| **Overall bias** | **Risk of bias judgement** | **Some concerns** | |

| | | | | | |
|---|---|---|---|---|---|
| **Unique ID** | Calabrò et. al. 2019 | **Study ID** | | **Assessor** | |
| **Ref or Label** | | **Aim** | assignment to intervention (the 'intention-to-treat' effect) | | |
| **Experimental** | | **Comparator** | | **Source** | Journal article(s) |
| **Outcome** | | **Results** | | **Weight** | 1 |

| Domain | Signalling question | Response | Comments |
|---|---|---|---|
| **Bias arising from the randomization process** | 1.1 Was the allocation sequence random? | PY | Random allocation to groups in a 1:1 ratio<br>Use of opaque sealed envelopes |
| | 1.2 Was the allocation sequence concealed until participants were enrolled and assigned to interventions? | Y | |
| | 1.3 Did baseline differences between intervention groups suggest a problem with the randomization process? | PN | |
| | **Risk of bias judgement** | **Low** | |
| **Bias due to deviations from intended interventions** | 2.1.Were participants aware of their assigned intervention during the trial? | PN | Random allocation to groups in a 1:1 ratio, insufficient details<br>Use of opaque sealed envelopes (prepared in advance and marked inside with signs + and) |
| | 2.2.Were carers and people delivering the interventions aware of participants' assigned intervention during the trial? | N | |
| | 2.3. If Y/PY/NI to 2.1 or 2.2: Were there deviations from the intended intervention that arose because of the experimental context? | NA | |
| | 2.4 If Y/PY to 2.3: Were these deviations likely to have affected the outcome? | NA | |
| | 2.5. If Y/PY/NI to 2.4: Were these deviations from intended intervention balanced between groups? | NA | |
| | 2.6 Was an appropriate analysis used to estimate the effect of assignment to intervention? | PY | |
| | 2.7 If N/PN/NI to 2.6: Was there potential for a substantial impact (on the result) of the failure to analyse participants in the group to which they were randomized? | NA | |
| | **Risk of bias judgement** | **Low** | |

| | | | |
|---|---|---|---|
| **Bias due to missing outcome data** | 3.1 Were data for this outcome available for all, or nearly all, participants randomized? | Y | No missing outcome data. |
| | 3.2 If N/PN/NI to 3.1: Is there evidence that result was not biased by missing outcome data? | NA | |
| | 3.3 If N/PN to 3.2: Could missingness in the outcome depend on its true value? | NA | |
| | 3.4 If Y/PY/NI to 3.3: Is it likely that missingness in the outcome depended on its true value? | NA | |
| | **Risk of bias judgement** | **Low** | |
| **Bias in measurement of the outcome** | 4.1 Was the method of measuring the outcome inappropriate? | N | |
| | 4.2 Could measurement or ascertainment of the outcome have differed between intervention groups? | PN | |
| | 4.3 Were outcome assessors aware of the intervention received by study participants? | N | |
| | 4.4 If Y/PY/NI to 4.3: Could assessment of the outcome have been influenced by knowledge of intervention received? | NA | |
| | 4.5 If Y/PY/NI to 4.4: Is it likely that assessment of the outcome was influenced by knowledge of intervention received? | NA | |
| | **Risk of bias judgement** | **Low** | |
| **Bias in selection of the reported result** | 5.1 Were the data that produced this result analysed in accordance with a pre-specified analysis plan that was finalized before unblinded outcome data were available for analysis? | PY | All pre-specified outcomes reported |
| | 5.2 ... multiple eligible outcome measurements (e.g. scales, definitions, time points) within the outcome domain? | PN | |
| | 5.3 ... multiple eligible analyses of the data? | PN | |
| | **Risk of bias judgement** | **Low** | |
| **Overall bias** | **Risk of bias judgement** | **Low** | |

| | | | | | |
|---|---|---|---|---|---|
| **Unique ID** | Braun Janzen et al. 2019 | **Study ID** | | **Assessor** | |
| **Ref or Label** | | **Aim** | assignment to intervention (the 'intention-to-treat' effect) | | |
| **Experimental** | | **Comparator** | | **Source** | Journal article(s) |
| **Outcome** | | **Results** | | **Weight** | 1 |

| **Domain** | **Signalling question** | **Response** | **Comments** |
|---|---|---|---|
| **Bias arising from the randomization process** | 1.1 Was the allocation sequence random? | Y | randomization.<br>No information. |
| | 1.2 Was the allocation sequence concealed until participants were enrolled and assigned to interventions? | NI | |
| | 1.3 Did baseline differences between intervention groups suggest a problem with the randomization process? | PN | |
| | **Risk of bias judgement** | **Some concerns** | |
| **Bias due to deviations from intended interventions** | 2.1.Were participants aware of their assigned intervention during the trial? | PN | Evaluators blinded to group designation |
| | 2.2.Were carers and people delivering the interventions aware of participants' assigned intervention during the trial? | N | |
| | 2.3. If Y/PY/NI to 2.1 or 2.2: Were there deviations from the intended intervention that arose because of the experimental context? | NA | |
| | 2.4 If Y/PY to 2.3: Were these deviations likely to have affected the outcome? | NA | |
| | 2.5. If Y/PY/NI to 2.4: Were these deviations from intended intervention balanced between groups? | NA | |
| | 2.6 Was an appropriate analysis used to estimate the effect of assignment to intervention? | PY | |
| | 2.7 If N/PN/NI to 2.6: Was there potential for a substantial impact (on the result) of the failure to analyse participants in the group to which they were randomized? | NA | |
| | **Risk of bias judgement** | **Low** | |
| **Bias due to missing outcome data** | 3.1 Were data for this outcome available for all, or nearly all, participants randomized? | PY | Only 4 participants out of 41 dropped out. |
| | 3.2 If N/PN/NI to 3.1: Is there evidence that result was not biased by missing outcome data? | NA | |
| | 3.3 If N/PN to 3.2: Could missingness in the outcome depend on its true value? | NA | |
| | 3.4 If Y/PY/NI to 3.3: Is it likely that missingness in the outcome depended on its true value? | NA | |
| | **Risk of bias judgement** | **Low** | |
| **Bias in measurement of the outcome** | 4.1 Was the method of measuring the outcome inappropriate? | PN | |
| | 4.2 Could measurement or ascertainment of the outcome have differed between intervention groups? | PN | |
| | 4.3 Were outcome assessors aware of the intervention received by study participants? | N | |
| | 4.4 If Y/PY/NI to 4.3: Could assessment of the outcome have been influenced by knowledge of intervention received? | NA | |
| | 4.5 If Y/PY/NI to 4.4: Is it likely that assessment of the outcome was influenced by knowledge of intervention received? | NA | |
| | **Risk of bias judgement** | **Low** | |
| **Bias in selection of the reported result** | 5.1 Were the data that produced this result analysed in accordance with a pre-specified analysis plan that was finalized before unblinded outcome data were available for analysis? | PY | All pre-specified outcomes reported. |
| | 5.2 ... multiple eligible outcome measurements (e.g. scales, definitions, time points) within the outcome domain? | PN | |
| | 5.3 ... multiple eligible analyses of the data? | PN | |
| | **Risk of bias judgement** | **Low** | |
| **Overall bias** | **Risk of bias judgement** | **Some concerns** | |

| | | | | | |
|---|---|---|---|---|---|
| **Unique ID** | Lee et al. 2018 | **Study ID** | | **Assessor** | |
| **Ref or Label** | | **Aim** | assignment to intervention (the 'intention-to-treat' effect) | | |
| **Experimental** | | **Comparator** | | **Source** | Journal article(s) |
| **Outcome** | | **Results** | | **Weight** | 1 |

| Domain | Signalling question | Response | Comments |
|---|---|---|---|
| **Bias arising from the randomization process** | 1.1 Was the allocation sequence random? | Y | Use of Random Allocation Software |
| | 1.2 Was the allocation sequence concealed until participants were enrolled and assigned to interventions? | PY | |
| | 1.3 Did baseline differences between intervention groups suggest a problem with the randomization process? | PN | |
| | **Risk of bias judgement** | **Low** | |
| **Bias due to deviations from intended interventions** | 2.1.Were participants aware of their assigned intervention during the trial? | PN | Evaluators blinded to group designation. |
| | 2.2.Were carers and people delivering the interventions aware of participants' assigned intervention during the trial? | Y | |
| | 2.3. If Y/PY/NI to 2.1 or 2.2: Were there deviations from the intended intervention that arose because of the experimental context? | PN | |
| | 2.4 If Y/PY to 2.3: Were these deviations likely to have affected the outcome? | NA | |
| | 2.5. If Y/PY/NI to 2.4: Were these deviations from intended intervention balanced between groups? | NA | |
| | 2.6 Was an appropriate analysis used to estimate the effect of assignment to intervention? | PY | |
| | 2.7 If N/PN/NI to 2.6: Was there potential for a substantial impact (on the result) of the failure to analyse participants in the group to which they were randomized? | NA | |
| | **Risk of bias judgement** | **Low** | |
| **Bias due to missing outcome data** | 3.1 Were data for this outcome available for all, or nearly all, participants randomized? | Y | Only 1 participants out of 45 dropped out.<br>All pre-specified outcomes reported. |
| | 3.2 If N/PN/NI to 3.1: Is there evidence that result was not biased by missing outcome data? | NA | |
| | 3.3 If N/PN to 3.2: Could missingness in the outcome depend on its true value? | NA | |
| | 3.4 If Y/PY/NI to 3.3: Is it likely that missingness in the outcome depended on its true value? | NA | |
| | **Risk of bias judgement** | **Low** | |
| **Bias in measurement of the outcome** | 4.1 Was the method of measuring the outcome inappropriate? | PN | |
| | 4.2 Could measurement or ascertainment of the outcome have differed between intervention groups? | PN | |
| | 4.3 Were outcome assessors aware of the intervention received by study participants? | N | |
| | 4.4 If Y/PY/NI to 4.3: Could assessment of the outcome have been influenced by knowledge of intervention received? | NA | |
| | 4.5 If Y/PY/NI to 4.4: Is it likely that assessment of the outcome was influenced by knowledge of intervention received? | NA | |
| | **Risk of bias judgement** | **Low** | |
| **Bias in selection of the reported result** | 5.1 Were the data that produced this result analysed in accordance with a pre-specified analysis plan that was finalized before unblinded outcome data were available for analysis? | PY | Use of Random Allocation Software<br>No information<br>Evaluators blinded to group designation |
| | 5.2 ... multiple eligible outcome measurements (e.g. scales, definitions, time points) within the outcome domain? | PN | |
| | 5.3 ... multiple eligible analyses of the data? | PN | |
| | **Risk of bias judgement** | **Low** | |
| **Overall bias** | **Risk of bias judgement** | **Low** | |

| | | | | | |
|---|---|---|---|---|---|
| **Unique ID** | Thaut et al. 2019 | **Study ID** | | **Assessor** | |
| **Ref or Label** | | **Aim** | assignment to intervention (the 'intention-to-treat' effect) | | |
| **Experimental** | | **Comparator** | | **Source** | Journal article(s) |
| **Outcome** | | **Results** | | **Weight** | 1 |

| Domain | Signalling question | Response | Comments |
|---|---|---|---|
| **Bias arising from the randomization process** | 1.1 Was the allocation sequence random? | Y | Use a computerized random selector program implemented by a computer specialist  external to the study to assure allocation concealment<br>Use a unique  computer-generated random |
| | 1.2 Was the allocation sequence concealed until participants were enrolled and assigned to interventions? | Y | |
| | 1.3 Did baseline differences between intervention groups suggest a problem with the randomization process? | N | |
| | **Risk of bias judgement** | **Low** | |
| **Bias due to deviations from intended interventions** | 2.1.Were participants aware of their assigned intervention during the trial? | PN | Evaluators blinded to group designation. |
| | 2.2.Were carers and people delivering the interventions aware of participants' assigned intervention during the trial? | N | |
| | 2.3. If Y/PY/NI to 2.1 or 2.2: Were there deviations from the intended intervention that arose because of the experimental context? | NA | |
| | 2.4 If Y/PY to 2.3: Were these deviations likely to have affected the outcome? | NA | |
| | 2.5. If Y/PY/NI to 2.4: Were these deviations from intended intervention balanced between groups? | NA | |
| | 2.6 Was an appropriate analysis used to estimate the effect of assignment to intervention? | PY | |
| | 2.7 If N/PN/NI to 2.6: Was there potential for a substantial impact (on the result) of the failure to analyse participants in the group to which they were randomized? | NA | |

| Domain | Signalling question | Response | Comments |
|---|---|---|---|
| | **Risk of bias judgement** | **Low** | |
| **Bias due to missing outcome data** | 3.1 Were data for this outcome available for all, or nearly all, participants randomized? | N | Missing outcome data balanced in numbers across intervention and control group, with similar reasons for missing data across |
| | 3.2 If N/PN/NI to 3.1: Is there evidence that result was not biased by missing outcome data? | N | |
| | 3.3 If N/PN to 3.2: Could missingness in the outcome depend on its true value? | PN | Missing outcome data balanced in numbers across intervention and control group, with similar reasons for missing data across groups |
| | 3.4 If Y/PY/NI to 3.3: Is it likely that missingness in the outcome depended on its true value? | NA | |
| | **Risk of bias judgement** | **Some concerns** | |
| **Bias in measurement of the outcome** | 4.1 Was the method of measuring the outcome inappropriate? | N | |
| | 4.2 Could measurement or ascertainment of the outcome have differed between intervention groups? | PN | |
| | 4.3 Were outcome assessors aware of the intervention received by study participants? | N | |
| | 4.4 If Y/PY/NI to 4.3: Could assessment of the outcome have been influenced by knowledge of intervention received? | NA | |
| | 4.5 If Y/PY/NI to 4.4: Is it likely that assessment of the outcome was influenced by knowledge of intervention received? | NA | |
| | **Risk of bias judgement** | **Low** | |
| **Bias in selection of the reported result** | 5.1 Were the data that produced this result analysed in accordance with a pre-specified analysis plan that was finalized before unblinded outcome data were available for analysis? | Y | Use a computerized random selector program implemented by a computer specialist, external to the study to assure |
| | 5.2 ... multiple eligible outcome measurements (e.g. scales, definitions, time points) within the outcome domain? | PN | |
| | 5.3 ... multiple eligible analyses of the data? | PN | |
| | **Risk of bias judgement** | **Low** | |
| **Overall bias** | **Risk of bias judgement** | **Some concerns** | |

| | | | | | |
|---|---|---|---|---|---|
| **Unique ID** | Murgia et al. 2018 | **Study ID** | | **Assessor** | |
| **Ref or Label** | | **Aim** | assignment to intervention (the 'intention-to-treat' effect) | | |
| **Experimental** | | **Comparator** | | **Source** | Journal article(s) |
| **Outcome** | | **Results** | | **Weight** | 1 |

| Domain | Signalling question | Response | Comments |
|---|---|---|---|
| **Bias arising from the randomization process** | 1.1 Was the allocation sequence random? | Y | Use of block randomisation generated by an online sequence generator (described in detail).<br><br>Described in sufficient detail to allow a |
| | 1.2 Was the allocation sequence concealed until participants were enrolled and assigned to interventions? | PY | |
| | 1.3 Did baseline differences between intervention groups suggest a problem with the randomization process? | PN | |
| | **Risk of bias judgement** | **Low** | |
| **Bias due to deviations from intended interventions** | 2.1.Were participants aware of their assigned intervention during the trial? | Y | Observer-blind trial. |
| | 2.2.Were carers and people delivering the interventions aware of participants' assigned intervention during the trial? | PY | |
| | 2.3. If Y/PY/NI to 2.1 or 2.2: Were there deviations from the intended intervention that arose because of the experimental context? | PN | |
| | 2.4 If Y/PY to 2.3: Were these deviations likely to have affected the outcome? | NA | |
| | 2.5. If Y/PY/NI to 2.4: Were these deviations from intended intervention balanced between groups? | NA | |
| | 2.6 Was an appropriate analysis used to estimate the effect of assignment to intervention? | PY | |
| | 2.7 If N/PN/NI to 2.6: Was there potential for a substantial impact (on the result) of the failure to analyse participants in the group to which they were randomized? | NA | |
| | **Risk of bias judgement** | **Low** | |
| **Bias due to missing outcome data** | 3.1 Were data for this outcome available for all, or nearly all, participants randomized? | N | Use of block randomisation generated by an online sequence generator (described in detail). |
| | 3.2 If N/PN/NI to 3.1: Is there evidence that result was not biased by missing outcome data? | PN | |
| | 3.3 If N/PN to 3.2: Could missingness in the outcome depend on its true value? | PN | |
| | 3.4 If Y/PY/NI to 3.3: Is it likely that missingness in the outcome depended on its true value? | NA | |
| | **Risk of bias judgement** | **Some concerns** | |
| **Bias in measurement of the outcome** | 4.1 Was the method of measuring the outcome inappropriate? | N | |
| | 4.2 Could measurement or ascertainment of the outcome have differed between intervention groups? | PN | |
| | 4.3 Were outcome assessors aware of the intervention received by study participants? | N | |
| | 4.4 If Y/PY/NI to 4.3: Could assessment of the outcome have been influenced by knowledge of intervention received? | NA | |
| | 4.5 If Y/PY/NI to 4.4: Is it likely that assessment of the outcome was influenced by knowledge of intervention received? | NA | |
| | **Risk of bias judgement** | **Low** | |
| **Bias in selection of the reported result** | 5.1 Were the data that produced this result analysed in accordance with a pre-specified analysis plan that was finalized before unblinded outcome data were available for analysis? | PY | All pre-specified outcomes reported. |
| | 5.2 ... multiple eligible outcome measurements (e.g. scales, definitions, time points) within the outcome domain? | PN | |
| | 5.3 ... multiple eligible analyses of the data? | PN | |
| | **Risk of bias judgement** | **Low** | |
| **Overall bias** | **Risk of bias judgement** | **Some concerns** | |

| | | | | | |
|---|---|---|---|---|---|
| **Unique ID** | Mainka et al. 2018 | **Study ID** | | **Assessor** | |
| **Ref or Label** | | **Aim** | assignment to intervention (the 'intention-to-treat' effect) | | |
| **Experimental** | | **Comparator** | | **Source** | Journal article(s) |
| **Outcome** | | **Results** | | **Weight** | 1 |

| Domain | Signalling question | Response | Comments |
|---|---|---|---|
| **Bias arising from the randomization process** | 1.1 Was the allocation sequence random? | Y | Use of block randomization (software randlist) by a<br>person not involved in the study.<br><br>Sealed, sequentially numbered envelopes |
| | 1.2 Was the allocation sequence concealed until participants were enrolled and assigned to interventions? | Y | |
| | 1.3 Did baseline differences between intervention groups suggest a problem with the randomization process? | PN | |
| | **Risk of bias judgement** | **Low** | |
| **Bias due to deviations from intended interventions** | 2.1.Were participants aware of their assigned intervention during the trial? | PN | Evaluators blinded to group designation. |
| | 2.2.Were carers and people delivering the interventions aware of participants' assigned intervention during the trial? | PN | |
| | 2.3. If Y/PY/NI to 2.1 or 2.2: Were there deviations from the intended intervention that arose because of the experimental context? | NA | |
| | 2.4 If Y/PY to 2.3: Were these deviations likely to have affected the outcome? | NA | |
| | 2.5. If Y/PY/NI to 2.4: Were these deviations from intended intervention balanced between groups? | NA | |
| | 2.6 Was an appropriate analysis used to estimate the effect of assignment to intervention? | PY | |
| | 2.7 If N/PN/NI to 2.6: Was there potential for a substantial impact (on the result) of the failure to analyse participants in the group to which they were randomized? | NA | |
| | **Risk of bias judgement** | **Low** | |
| **Bias due to missing outcome data** | 3.1 Were data for this outcome available for all, or nearly all, participants randomized? | N | Of the 45 participants, 10 dropped out and the reasons were described. Three groups of almost equal numbers took part in the |
| | 3.2 If N/PN/NI to 3.1: Is there evidence that result was not biased by missing outcome data? | N | |
| | 3.3 If N/PN to 3.2: Could missingness in the outcome depend on its true value? | PN | |
| | 3.4 If Y/PY/NI to 3.3: Is it likely that missingness in the outcome depended on its true value? | NA | |
| | **Risk of bias judgement** | **Some concerns** | |
| **Bias in measurement of the outcome** | 4.1 Was the method of measuring the outcome inappropriate? | N | |
| | 4.2 Could measurement or ascertainment of the outcome have differed between intervention groups? | PN | |
| | 4.3 Were outcome assessors aware of the intervention received by study participants? | N | |
| | 4.4 If Y/PY/NI to 4.3: Could assessment of the outcome have been influenced by knowledge of intervention received? | NA | |
| | 4.5 If Y/PY/NI to 4.4: Is it likely that assessment of the outcome was influenced by knowledge of intervention received? | NA | |
| | **Risk of bias judgement** | **Low** | |
| **Bias in selection of the reported result** | 5.1 Were the data that produced this result analysed in accordance with a pre-specified analysis plan that was finalized before unblinded outcome data were available for analysis? | Y | The study protocol is available; all pre-specified outcomes reported. |
| | 5.2 ... multiple eligible outcome measurements (e.g. scales, definitions, time points) within the outcome domain? | PN | |
| | 5.3 ... multiple eligible analyses of the data? | PN | |
| | **Risk of bias judgement** | **Low** | |
| **Overall bias** | **Risk of bias judgement** | **Some concerns** | |

| | | | | | |
|---|---|---|---|---|---|
| **Unique ID** | Bella et al. 2017 | **Study ID** | | **Assessor** | |
| **Ref or Label** | | **Aim** | assignment to intervention (the 'intention-to-treat' effect) | | |
| **Experimental** | | **Comparator** | | **Source** | Journal article(s) |
| **Outcome** | | **Results** | | **Weight** | 1 |

| Domain | Signalling question | Response | Comments |
|---|---|---|---|
| **Bias arising from the randomization process** | 1.1 Was the allocation sequence random? | NI | No information. |
| | 1.2 Was the allocation sequence concealed until participants were enrolled and assigned to interventions? | NI | |
| | 1.3 Did baseline differences between intervention groups suggest a problem with the randomization process? | PN | |
| | **Risk of bias judgement** | **Some concerns** | |
| **Bias due to deviations from intended interventions** | 2.1.Were participants aware of their assigned intervention during the trial? | NI | No information. |
| | 2.2.Were carers and people delivering the interventions aware of participants' assigned intervention during the trial? | NI | |
| | 2.3. If Y/PY/NI to 2.1 or 2.2: Were there deviations from the intended intervention that arose because of the experimental context? | PN | |
| | 2.4 If Y/PY to 2.3: Were these deviations likely to have affected the outcome? | NA | |
| | 2.5. If Y/PY/NI to 2.4: Were these deviations from intended intervention balanced between groups? | NA | |
| | 2.6 Was an appropriate analysis used to estimate the effect of assignment to intervention? | NI | |

| | | | |
|---|---|---|---|
| | 2.7 If N/PN/NI to 2.6: Was there potential for a substantial impact (on the result) of the failure to analyse participants in the group to which they were randomized? | NI | |
| | **Risk of bias judgement** | **High** | |
| **Bias due to missing outcome data** | 3.1 Were data for this outcome available for all, or nearly all, participants randomized? | N | Of the 21 person intervention group, 7 subjects dropped out (reasons described). Controls' performance was assessed only [illegible] |
| | 3.2 If N/PN/NI to 3.1: Is there evidence that result was not biased by missing outcome data? | N | |
| | 3.3 If N/PN to 3.2: Could missingness in the outcome depend on its true value? | PN | |
| | 3.4 If Y/PY/NI to 3.3: Is it likely that missingness in the outcome depended on its true value? | NA | |
| | **Risk of bias judgement** | **Some concerns** | |
| **Bias in measurement of the outcome** | 4.1 Was the method of measuring the outcome inappropriate? | PN | |
| | 4.2 Could measurement or ascertainment of the outcome have differed between intervention groups? | PN | |
| | 4.3 Were outcome assessors aware of the intervention received by study participants? | PN | |
| | 4.4 If Y/PY/NI to 4.3: Could assessment of the outcome have been influenced by knowledge of intervention received? | NA | |
| | 4.5 If Y/PY/NI to 4.4: Is it likely that assessment of the outcome was influenced by knowledge of intervention received? | NA | |
| | **Risk of bias judgement** | **Low** | |
| **Bias in selection of the reported result** | 5.1 Were the data that produced this result analysed in accordance with a pre-specified analysis plan that was finalized before unblinded outcome data were available for analysis? | PY | All pre-specified outcomes reported. |
| | 5.2 ... multiple eligible outcome measurements (e.g. scales, definitions, time points) within the outcome domain? | PN | |
| | 5.3 ... multiple eligible analyses of the data? | PN | |
| | **Risk of bias judgement** | **Low** | |
| **Overall bias** | **Risk of bias judgement** | **High** | |
